# Time Course of Central and Peripheral Alterations after Isometric Neuromuscular Electrical Stimulation-Induced Muscle Damage

**DOI:** 10.1371/journal.pone.0107298

**Published:** 2014-09-12

**Authors:** Alexandre Fouré, Kazunori Nosaka, Jennifer Wegrzyk, Guillaume Duhamel, Arnaud Le Troter, Hélène Boudinet, Jean-Pierre Mattei, Christophe Vilmen, Marc Jubeau, David Bendahan, Julien Gondin

**Affiliations:** 1 Aix-Marseille University, CNRS, CRMBM UMR CNRS 7339, Marseille, France; 2 Edith Cowan University, School of Exercise and Health Sciences, WA 6027, Joondalup, Australia; 3 APHM, La Timone Hospital, CEMEREM, Imaging Center, Marseille, France; 4 APHM, La Conception Hospital, Department of Rheumatology, Marseille, France; 5 University of Nantes, Laboratory “Motricité, Interactions, Performance” (EA 4334), UFR STAPS, Nantes, France; University of Alberta, Canada

## Abstract

Isometric contractions induced by neuromuscular electrostimulation (NMES) have been shown to result in a prolonged force decrease but the time course of the potential central and peripheral factors have never been investigated. This study examined the specific time course of central and peripheral factors after isometric NMES-induced muscle damage. Twenty-five young healthy men were subjected to an NMES exercise consisting of 40 contractions for both legs. Changes in maximal voluntary contraction force of the knee extensors (MVC), peak evoked force during double stimulations at 10 Hz (Db_10_) and 100 Hz (Db_100_), its ratio (10∶100), voluntary activation, muscle soreness and plasma creatine kinase activity were assessed before, immediately after and throughout four days after NMES session. Changes in knee extensors volume and T_2_ relaxation time were also assessed at two (D2) and four (D4) days post-exercise. MVC decreased by 29% immediately after NMES session and was still 19% lower than the baseline value at D4. The decrease in Db_10_ was higher than in Db_100_ immediately and one day post-exercise resulting in a decrease (−12%) in the 10∶100 ratio. On the contrary, voluntary activation significantly decreased at D2 (−5%) and was still depressed at D4 (−5%). Muscle soreness and plasma creatine kinase activity increased after NMES and peaked at D2 and D4, respectively. T_2_ was also increased at D2 (6%) and D4 (9%). Additionally, changes in MVC and peripheral factors (e.g., Db_100_) were correlated on the full recovery period, while a significant correlation was found between changes in MVC and VA only from D2 to D4. The decrease in MVC recorded immediately after the NMES session was mainly due to peripheral changes while both central and peripheral contributions were involved in the prolonged force reduction. Interestingly, the chronological events differ from what has been reported so far for voluntary exercise-induced muscle damage.

## Introduction

Neuromuscular electrical stimulation (NMES) activates intramuscular nerve branches *via* surface electrodes placed over a muscle thereby evoking muscle contractions [1]. Although NMES is commonly used in the fields of training and rehabilitation as a tool for improving, preserving or restoring muscle functional capacities [2]–[4], recent studies have provided compelling evidence of adverse effects [5]. Histological analyses clearly showed macrophage infiltration, z-lines disruption and a slightly modified desmin staining [6], [7]. In addition, a high satellite cell content and increases in types I and III collagens have been reported after NMES [8]. Under isometric conditions [9], [10], NMES has been linked to a 10- to 30-fold increase in creatine kinase (CK) activity further confirming the damaging effects. In addition to muscle tissue alterations, a prolonged decrease in force production ability of approximately 25% has been measured for several days following a single bout of NMES isometric exercise [9], [10].

From a general point of view, the reduced muscle function resulting from a voluntary eccentric exercise-induced muscle damage has been typically related to peripheral (i.e., below the neuromuscular junction) and/or central (i.e., spinal and/or supraspinal) factors. Peripheral factors have been commonly linked to an altered excitation-contraction coupling [11] due to, among other, a decreased intracellular calcium concentration or a reduced sensitivity of the myofilaments to calcium [12] leading to diminished maximal evoked force production [13]. The latter phenomenon has been related to changes in muscle structure including z-line disruptions through analysis of biopsy samples [6], [7], alterations of muscle mechanical properties [14], [15], edema and inflammation phenomena identified by the increase in transverse relaxation time (T_2_) with magnetic resonance imaging [16]. Interestingly, it has been suggested that the potential activation of group III and IV muscle afferents by inflammatory mediators could impair neural drive [17]–[19]. Accordingly, central adaptations occurring after voluntary eccentric exercise-induced muscle damage have been disclosed on the basis of decreased voluntary activation [13], [20].

The immediate force reduction after a NMES exercise has been mainly related to peripheral alterations considering that voluntary activation was unchanged at least for knee extensor muscles [21], [22]. The accounting factors of the prolonged force loss after isometric NMES have been poorly investigated and the respective contributions of central and peripheral mechanisms have never been determined.

Interestingly, the MVC time-dependent changes resulting from damaging exercise using isometric NMES are comparable to what has been reported for a voluntary eccentric exercise [5]. The corresponding accounting peripheral and central factors have been well documented after voluntary eccentric exercise-induced muscle damage [13], [20], [23], [24]. The voluntary activation level has been reported to be reduced immediately after the eccentric exercise session and to fully recover within the first two days post-exercise [13], [23]. On the contrary, voluntary activation has been reported to be unchanged immediately after a single NMES session [21], [22] and a delayed (i.e., 2 days after NMES exercise) reduction has been suggested on the basis of surface electromyographic measurements [25]. However, the involvement of peripheral mechanisms potentially accounting for the voluntary activation loss, suggested in a delayed time after an isometric NMES exercise, has not been investigated.

The aim of the present study was to assess the time course and the contribution of both peripheral and central factors to force decrease resulting from a single bout of isometric NMES exercise of the knee extensor muscles. We combined biochemical, magnetic resonance imaging and electrophysiological measurements in order to investigate the potential interaction between muscular (i.e., changes in muscle volume, T_2_ and contractile properties) and neural (i.e., change in voluntary activation) modulations associated to the functional alterations (i.e., the decrease in maximal voluntary contraction force) resulting from the isometric NMES-induced muscle damage. We hypothesized that both central and peripheral factors would be involved with a specific time course in the prolonged force loss associated to NMES.

## Materials and Methods

### Subjects

Twenty-five healthy men (age: 22 (2) years, height: 178 (6) cm, body mass: 68 (7) kg) volunteered to participate in this study. Subjects were recreationally active (exercise time: 36 (42) min.wk^−1^) but none of them were engaged in any training or regular exercise programs. They were instructed to avoid any intense and non-familiar physical activities throughout the duration of the protocol. Subjects were asked to keep their diet habits and to limit their alcohol consumption throughout the study period. They were instructed to avoid caffeine consumption and smoking before the experiments. Consumption of medicines was prohibited from seven days before to five days after the NMES session.

### Ethics statement

Subjects were fully informed about the nature and the aim of the study and gave their written informed consent to participate. The study was approved by the Local Human Research Ethics Committee Sud Mediterranée V (n° 2012-04 A00449-34), and was conducted in conformity with the Declaration of Helsinki.

### Study design

Assessments of maximal voluntary contraction (MVC) force of knee extensors, muscle soreness, plasma CK activity and neuromuscular variables were performed about one hour before (i.e., Baseline), immediately after (AFTER) and one (D1), two (D2), three (D3) and four days (D4) after the NMES session. All the testing sessions were performed at the same time of the day. In addition, T_2_ relaxation time and the *quadriceps femoris* (QF) muscles volume were measured by magnetic resonance imaging at six days before NMES exercise (D-6), D2 and D4. We shed light on the time course of neuromuscular alteration in the first four days following an isometric NMES EIMD. We have been unable to investigate the recovery of the measured variables over a longer time period after the NMES because of logistical constraints. However the variables measured in the present study have been shown to completely recover within weeks after a voluntary EIMD [13], [25]–[28].

### NMES session

Subjects were seated on a specific chair (Multi-Form', La Roque d'Anthéron, France) equipped with a calibrated force sensor. Hip and ankle joints were secured by adjustable lap belts to hold the hip and knee angles at ∼90° and ∼100° respectively (0° corresponding to the full extension). Both legs were stimulated simultaneously using three stimulation electrodes placed over the thigh skin. A 5 cm×10 cm electrode was positioned on the proximal part of the thigh (i.e., placed ∼5 cm below the inguinal ligament) and two 5 cm×5 cm electrodes were located on the *vastus lateralis* [VL] and *vastus medialis* [VM] muscle bellies [29]. Biphasic symmetric rectangular pulses were delivered at a frequency of 100 Hz using a portable battery-powered stimulator (Compex Performance, Djo Global, France). Pulse duration was 400 µs (40 contractions, duty cycle = 12.5% with 5 s on and 35 s off throughout the NMES exercise) and stimulation intensity was gradually increased in order to reach the maximal level of evoked force according to the pain threshold (i.e., level of maximal tolerance) of each subject similarly to previous studies [9], [10], [30].

Force signal was sampled at 1000 Hz using a Powerlab system and Labchart software (ADinstruments, Colorado Springs, CO, USA) and stored for analysis with Matlab software (v7.11, Mathworks, Natick, MA, USA). Peak force was measured for each NMES-evoked contraction, visualized in real time by experimenter and *a posteriori* averaged over the forty contractions.

### Maximal voluntary isometric contraction (MVC) force

Subjects were seated on the chair with the knee flexed at ∼100° and performed a 5–10 minutes warm-up session including a set of sub-maximal bilateral isometric knee extensions. Subjects were then instructed to perform three bilateral isometric MVCs of the knee extensors and three unilateral isometric MVCs with the right leg (MVC_right_). A resting period of at least 30 s separated each MVC. MVC_right_ were determined as the highest value among the three trials.

### Neuromuscular parameters

Transcutaneous femoral nerve stimulation was used to evoke isometric contractions of the right knee extensors. Electrical stimulation was applied percutaneously at the motor nerve level *via* a self-adhesive electrode (10-mm diameter, Ag-AgCl, Asept Inmed, Quint Fonsegrives, France) pressed and maintained manually, by the same experimenter for all neuromuscular measurements, in the femoral triangle, 3–5 cm below the inguinal ligament. The anode using a 10 cm×5 cm self-adhesive stimulation electrode (Stimex, Wetzlar, Germany), was located in the gluteal fold. Rectangular pulses (1 ms duration, 400 V maximal voltage) were delivered by a constant current stimulation unit (Digitimer DS7, Welwyn Garden City, UK). Current intensity was progressively increased by 2 mA increments until there was no further increase in peak twitch force. This current intensity, which was determined at the beginning of each testing session, was further increased by 20% (i.e., supramaximal) and then maintained for the entire testing procedure.

The testing procedure was composed of a pair of stimuli (100 Hz, 10-ms interstimulus interval) delivered over the isometric plateau of unilateral MVC (superimposed doublet). After (∼5 s later – i.e., automatic timing), this superimposed stimulation, a set of potentiated high-frequency doublet (100 Hz, 10-ms interstimulus interval, Db_100_), low frequency doublet (10 Hz, 100-ms interstimulus interval, Db_10_) and single twitch (Tw) was delivered to the relaxed muscles [31] with a constant time interval of 5 s (i.e., automatic timing). The whole testing procedure including superimposed stimulation and evoked contractions on resting muscle was performed at least three times.

External output trigger from the electrostimulator and force signals were simultaneously recorded in order to automatically characterize peak force for single twitch and doublets using a custom Matlab routine. Time to peak force (TTP) was determined at rest for Db_100_ and was defined relative to the onset of force (i.e., the first point with a value higher than the mean value calculated over the resting state). Rate of force development (RFD) was determined as the slope of the force-time relationship for Db_100_ from the onset of force generation to the peak force. The ratio between peak force associated to Db_10_ and Db_100_ (10∶100) was also calculated and used to assess prolonged low frequency force depression [32]. For these neuromuscular parameters, the average value computed from the best three sets was considered.

VA, representing the ability to voluntarily activate the QF muscles, was assessed according to the twitch interpolation technique [33] and calculated using the modified following equation provided by Strojnik and Komi [34] and used in several previous studies [13], [23]:
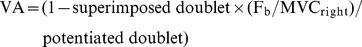
where F_b_ represents the force determined before the superimposed doublet. F_b_ was determined as the mean value of force developed during the 500 ms window before the superimposed 100 Hz doublet. The amplitude of the superimposed doublet was measured over a 200 ms time window after the signal trigger from the stimulator corresponding to the superimposed 100 Hz doublet.

### Muscle soreness

Level of muscle soreness was assessed during a squat contraction, passive stretching of knee joint and palpation of VL and VM (knee angle: ∼100°) using visual analog scale (VAS) with a 100 mm horizontal line with “no pain” on the one end (0 mm) and “extremely painful” on the other (100 mm). Subjects were asked to mark their pain level on the VAS for each modality and scores were averaged for each subject. Squat movement was performed from standing position to a knee flexion angle of 90° with a constant movement velocity (i.e., 2 s for movement from standing position) for all session and subjects. The final squat position was maintained during 3 s. Each test modality to determine muscle soreness was performed by the same investigator. Soreness was assessed using the mean value obtained from the measurements on the four measures (i.e., isometric squat, passive stretching, VL and VM palpation).

### Plasma CK activity

Blood was drawn from the forearm vein of each subject (5 mL) following a standardized procedure. Plasma was immediately separated after blood centrifugation (15 min at 2500 rpm). Plasma CK activity was determined enzymatically at 37°C using a chemistry analyzer (UniCel DxC 800 Synchron Clinical System; Beckman Coulter, Inc., Brea, CA, USA).

### Magnetic resonance imaging

Subjects lay supine with the right leg centered within a 1.5-T super-conducting magnet (MAGNETOM Avanto, Siemens AG, Healthcare Sector, Erlangen, Germany). The 6-channels flexible surface body coil (Siemens AG, Healthcare Sector, Erlangen, Germany) was placed around the right thigh muscles. QF muscles volume was determined from high-resolution T_1_-weighted images (20 slices, field of view (FOV) = 220×220 mm^2^; matrix = 576×576; TR = 549 ms; TE = 13 ms; Number of repetitions (N_EX_) = 1; slice thickness = 6 mm; gap between slices = 6 mm, acquisition time = 5 min 18 s). T_2_-weighted images were acquired with a segmented (15 segments) echo planar imaging sequence with TE = 15, 25, 35, 45 and 55 ms. Other parameters were 20 slices, FOV = 220×220 mm^2^; matrix = 192×192; TR = 4800 ms; N_EX_ = 1; slice thickness = 6 mm; gap between slices = 6 mm, Short TI Inversion Recovery (STIR) for fat saturation; acquisition time = 5 min 10 s. The most distal T_1_- and T_2_-weighted images were acquired at approximately 20 mm (i.e., 5% of the thigh length measured for each subject) upper the proximal border of the patella. The proposed technique combines the advantages of being low-sensitive to B_1_ inhomogeneities and based on the multiple Hahn echoes strategy. Segmented SE-EPI was previously validated on a phantom and *in vivo* for T_2_ measurements in exercising muscle [35]. It is noteworthy that TE range used for T_2_ determination could appear narrow as compared with that of other studies [36], [37]. However, resting T_2_ values of healthy thigh muscles are around 30–35 ms [38], [39] and expected to increase in a range between 35 and 45 ms in the days following the damaging [40]. In addition, the linear fit applied on data provided an accurate estimation of slightly higher T_2_ values. However, for much higher expected T_2_ values, higher values of TE would have been required.

Images were analyzed with FSL (FMRIB, Oxford, USA). A region of interest (ROI) was drawn in each slice by manually tracing the boundaries of the QF muscles anatomic cross-sectional area (ACSA). Using the truncated cone formula [41], QF muscles volume was calculated by summing the areas of all the slices, taking into account the slice thickness and the gaps between slices.

T_2_ maps were generated by a linear fit on a pixel-by-pixel basis the logarithm of the data to the following equation:
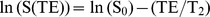
where S(TE) is the signal at time equal to TE and S_0_ is the equilibrium magnetization. Thereafter, T_2_ maps were resized to T_1_-weighted images definition. Then, ROIs initially drawn on T_1_-weighted images were used to analyze T_2_ maps and determine a mean T_2_ value for the whole QF muscles.

### Reproducibility of the measurements

Two pilot studies were conducted to assess the reproducibility of the measured parameters. The first study was performed in twenty four male subjects (22 (2) years, 178 (6) cm, 69 (8) kg) to assess intraclass correlation coefficient (ICC), standard error of the mean (SEM) and coefficient of variation (CV) for bilateral MVC, MVC_right_, peak force of Db_100_ and Tw, TTP and RFD for Db_100_, VA and 10∶100 ratio. The second study was conducted on seven healthy men (32 (6) years, 178 (7) cm, 72 (10) kg) to assess the reproducibility of magnetic resonance imaging parameters (i.e., QF muscles volume and T_2_). For both pilot studies, two testing sessions were separated by a two-day resting period, were performed at the same time of the day. ICC (2,1), SEM and CV showed a high reproducibility for both neuromuscular and magnetic resonance imaging (MRI) parameters as illustrated by the low CV (<9.6%) and high ICC (>0.76) values (Table 1).

**Table 1:** Day to day reliability for neuromuscular and magnetic resonance imaging parameters.

	Test 1	Test 2	SEM	CV (%)	ICC	Lower and upper CL
**MVC_right_ (N)**	333 (65)	337 (71)	21	6.3	0.90	(0.82–0.95)
**Tw (N)**	77 (19)	80 (19)	6	8.8	0.89	(0.75–0.95)
**Db_10_ (N)**	116 (26)	118 (26)	9	9.6	0.88	(0.75–0.95)
**Db_100_ (N)**	136 (25)	134 (26)	6	4.6	0.94	(0.86–0.97)
**10∶100**	0.85 (0.12)	0.88 (0.14)	0.05	7.8	0.84	(0.67–0.93)
**VA (%)**	95.0 (3.9)	94.8 (4.0)	1.9	2.2	0.76	(0.58–0.88)
**TTP (ms)**	78 (7)	76 (7)	3	3.9	0.82	(0.63–0.92)
**RFD (N/s)**	2119 (383)	2099 (383)	137	6.8	0.87	(0.73–0.94)
**QF Volume (cm^3^)**	1600 (294)	1616 (316)	20	1.1	1.00	(0.98–1.00)
**QF T_2_ (ms)**	33.5 (0.8)	33.0 (0.8)	0.2	0.5	0.96	(0.81–0.99)

SD: standard deviation, SEM: standard error of the mean, CV: coefficient of variation, ICC: intra-class correlation, CL: confidence limits.

MVC_right_: right leg maximal voluntary isometric contraction force, Tw: evoked force by a single twitch stimulation Db_10_: evoked force by a doublet at 10 Hz, Db_100_: evoked force by a doublet at 100 Hz, 10∶100: ratio Db_10_/Db_100_, VA: voluntary activation, TTP: time-to-peak force during 100 Hz doublet, RFD: maximal rate of force development during 100 Hz doublet, QF: quadriceps femoris.

### Statistics

After checking the distribution of data using Shapiro-Wilk test, parametric statistical tests were performed using Statistica software (Statsoft, Tulsa, OK). One-way repeated measures ANOVA were performed to assess changes in MVC_right_, muscle soreness, plasma CK activity, neuromuscular parameters and magnetic resonance imaging data. A Tukey's HSD post hoc analysis was conducted when appropriate. To assess the correlation among measured parameters, both the Pearson product-moment correlation (r) and Spearman rank correlation (ρ) coefficients were computed. For correlation analyses, changes from baseline were considered for all the parameters. Considering the high number of variables and the time point of measurements (i.e., after, D1, D2, D3 and D4), the correlations were reported into three different matrices. Correlations between changes in neuromuscular parameters in the early phase of recovery by taking into account both the measurements performed immediately after NMES and at D1 were firstly reported. The same variables in the delayed phase of recovery, including measurements performed at D2, D3 and D4, were then described. Finally, changes in MRI parameters and VA, MVC_right_, Db_100_ and plasma CK activity were included in a third matrix. The level of significance was set at P<0.05. Results are reported as mean (SD).

## Results

### NMES session

Stimulation intensity was gradually increased throughout the NMES session from 33 (6) mA to 65 (16) mA at the 40^th^ contraction. The averaged peak evoked force over the forty contractions was 255 (59) N corresponding to 32 (7) % of the bilateral MVC. Electrically-evoked force increased in the first 15 contractions and then plateaued after the 20^th^ contraction despite the continuous increase in stimulation intensity.

### MVC force

**Figure 1:**
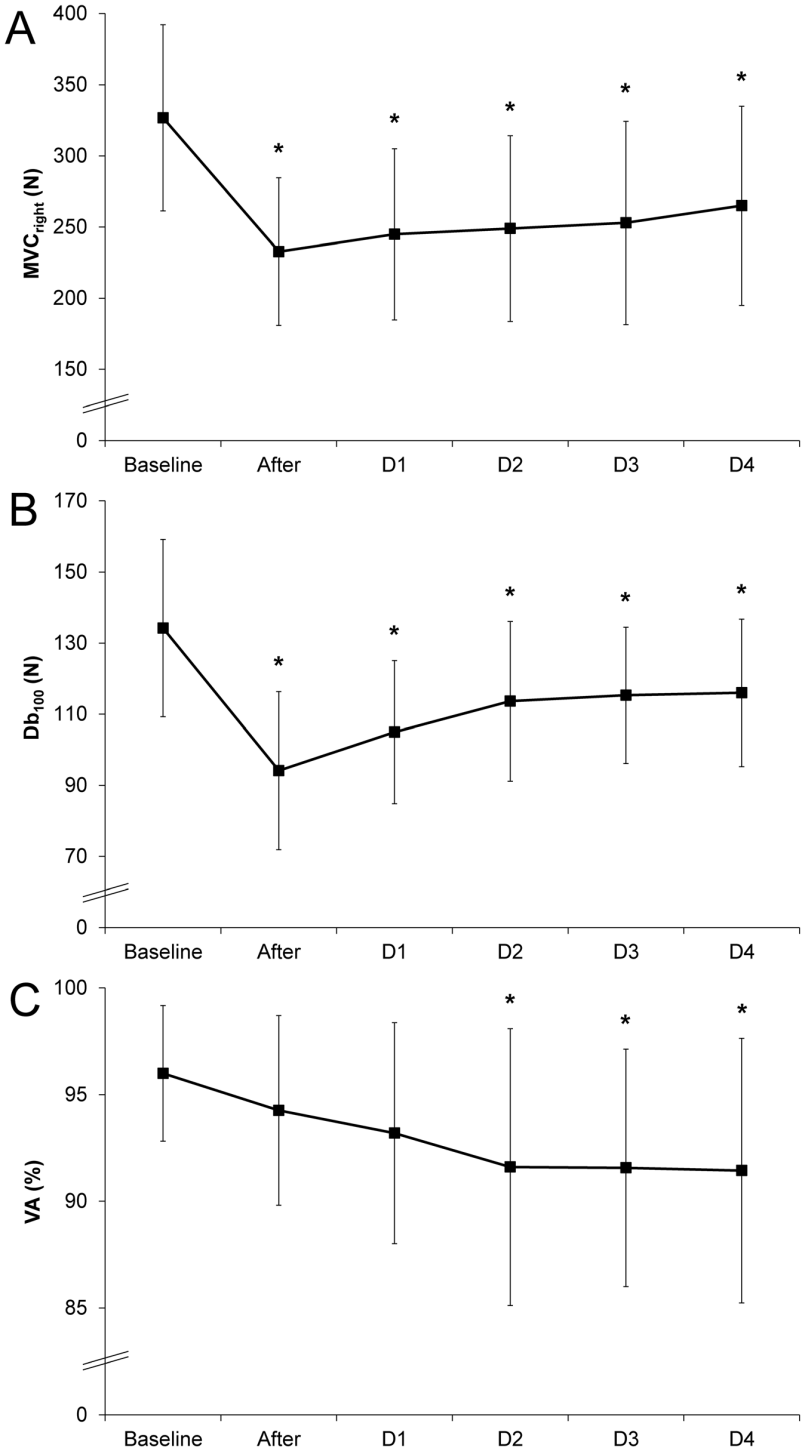
Time course of indirect markers of muscle damage before and after the isometric NMES session. Maximal voluntary contraction force (MVC_right_) performed with the right leg (A), force produced by a 100 Hz double stimuli applied on femoral nerve on the resting muscles (B) and voluntary activation (VA) during a maximal isometric voluntary contraction (C) before (Baseline), immediately after and 1, 2, 3 and 4 days (D1, D2, D3 and D4, respectively) after a single bout of neuromuscular electrical stimulation. Error bars represent standard deviation. *Significantly different from Baseline (P<0.05).

MVC_right_ decreased by -28% immediately after NMES session and was still lower than the baseline at D1 (−25%), D2 (−23%), D3 (−21%) and D4 (−18%) (Figure 1-A).

### Neuromuscular parameters

**Figure 2:**
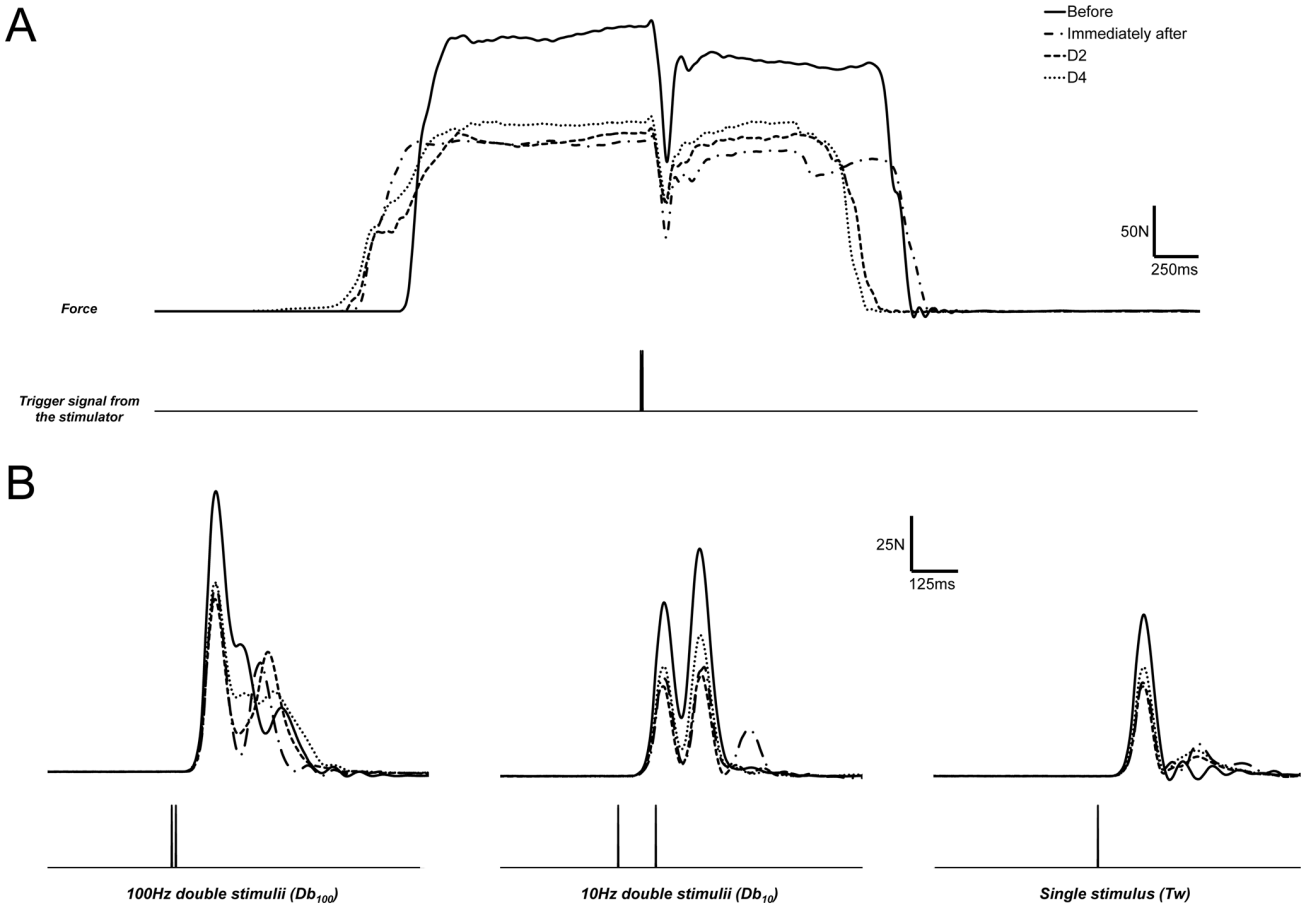
Mechanical data obtained during superimposed stimulations and evoked contractions on resting muscle. Typical raw data in a subject for the assessment of neuromuscular parameters including: (A) superimposed 100 Hz double stimuli during unilateral maximal voluntary contraction plateau and, (B) measurements on resting muscle: 100 Hz double stimuli (Db_100_), 10 Hz double stimuli (Db_10_) and single stimulus (Tw) before, immediately after, two and four days (D2 and D4, respectively) after the isometric NMES session. Bottom trace for each panel displays the output trigger signal from the electrostimulator.

A typical example of raw data obtained during the assessment of neuromuscular parameters on one representative subject is displayed in Figure 2. As shown in Figure 1-B, the Db_100_ peak force decreased by 30% immediately after the NMES session and remained below the baseline value at D1 (−20%), D2 (−13%), D3 (−12%) and D4 (−10%). A similar time course was recorded for Db_10_ and single twitch peak force with a large decrease (−38% and −36%) immediately after the NMES bout and at D1 (−31% and −30%), and a slighter decrease at D2 (−16% and −13%), D3 (−14% and −11%) and D4 (−10% and −12%). Interestingly, the 10∶100 ratio decreased significantly (−12%) immediately after the NMES bout and at D1 (13%) but totally recovered towards the initial value at D2 (Table 2). No significant TTP change was observed (P>0.05) throughout the NMES session whereas RFD significantly decreased immediately after the NMES bout (−28%) and slightly recovered at D1 (−20%). It was still diminished at D2 (−11%) and D3 (-12%) (Table 2).

**Table 2:** Muscle soreness (VAS), plasma creatine kinase activity and neuromuscular parameters recorded before (Baseline), immediately after and at D1, D2, D3 and D4 after the isometric NMES session.

	Baseline	After	D1	D2	D3	D4
**VAS score (mm)**	5 (4)	3 (3)	6 (6)	12 (9)	16 (9)	14 (9)
**Plasma CK activity (IU/L)**	171 (136)	163 (113)	413 (277)	1848 (2191)	7227 (7499)	12460 (17206)
**Tw (N)**	73 (16)	48 (17)^a^ ^,b^	51 (16)^a^ ^,b^	65 (15)	66 (14)	68 (12)
**Db_10_ (N)**	110 (21)	70 (21)^a^ ^,b^	76 (19)^a^ ^,b^	93 (18)^a^	95 (14)^a^	98 (17)^a^
**10∶100**	0.86 (0.12)	0.76 (0.15)^a^ ^,b^	0.75 (0.13)^a^ ^,b^	0.84 (0.14)	0.85 (0.12)	0.86 (0.13)
**TTP (ms)**	78 (7)	81 (14)	75 (9)	78 (10)	78 (8)	76 (8)
**RFD (N/s)**	1991 (255)	1442 (343)^a^ ^,b^	1589 (275)^a^	1766 (284)^a^	1736 (206)^a^	1815 (265)

a Significantly different from baseline (P<0.05); ^b^significantly different from D2, D3 and D4 (P<0.05).

VAS: score in visual analog scale, CK: plasma creatine kinase activity, Tw: evoked force by a single twitch stimulation Db_10_: evoked force by a doublet at 10 Hz, Db_100_: evoked force by a doublet at 100 Hz, 10∶100: ratio Db_10_/Db_100_, TTP: time-to-peak force during 100 Hz doublet, RFD: maximal rate of force development during 100 Hz doublet.

VA changes were delayed with respect to the NMES session (Figure 1-C). While no significant change in VA was found immediately after NMES exercise and at D1, a significant decrease was measured at D2 (91.6 (6.5) %), D3 (91.6 (5.6) %) and D4 (91.4 (6.2) %) as compared to baseline (96.0 (3.2) %).

### Muscle soreness

Muscle soreness significantly increased at D1, reached its maximum value at D2 and was still higher than the baseline value at D3 (Table 2). Changes in VAS score were similar among the test modalities.

### Plasma CK activity

We observed a large variability among subjects for plasma CK activity changes following NMES session. Plasma CK activity was unchanged at D1 and D2, significantly increased at D3 and further up at D4 (Table 2). This latter increase amounted to 70 times the baseline level (i.e., from 171 (135) IU.L^−1^ [range: 63–549]) to 12460 (17206) IU.L^−1^ [range: 1350–85000]).

### Magnetic resonance imaging

**Figure 3:**
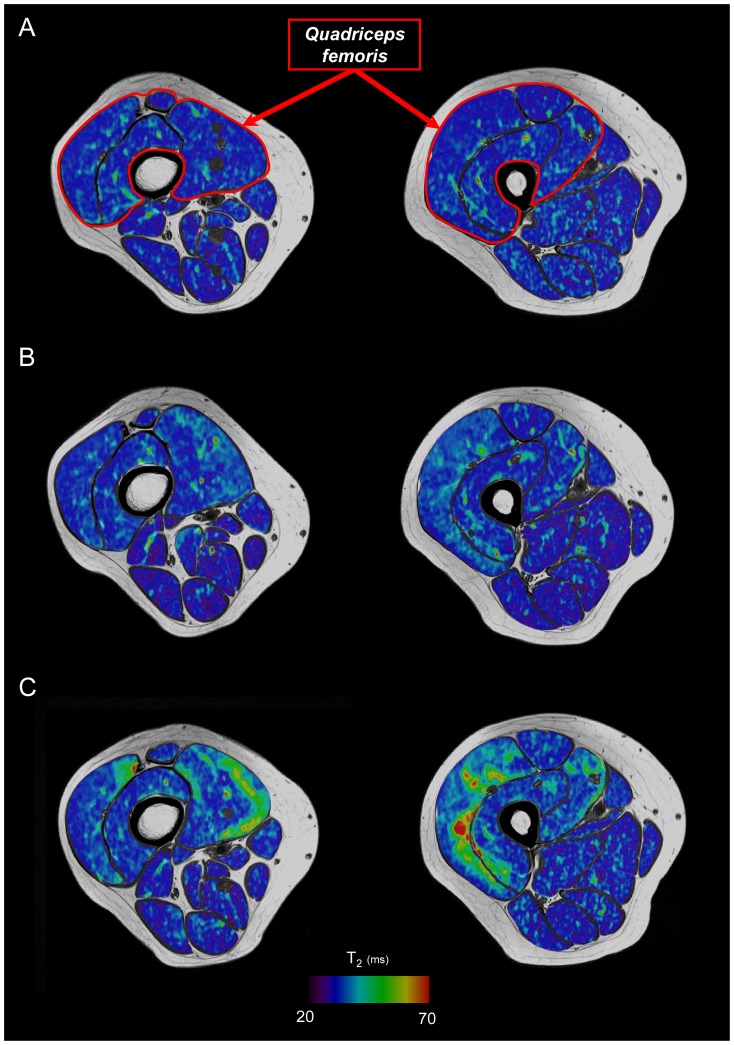
Changes in T_2_ maps color due to muscle alterations resulting from the damaging exercise. Axial T_2_ maps superimposed on T_1_-weighted images acquired from the distal (left) and proximal (right) regions of the thigh for a representative subject before (A), two days (B) and four days (C) after the isometric NMES session.

As shown in Table 3, both QF muscles volume and T_2_ significantly increased at D2 (+4% and +6%, respectively) and further increased at D4 (+5% and +9%, respectively) (Figure 3).

**Table 3:** Muscle volume and T_2_ values measured at D-6, D2 and D4 after the isometric NMES session.

	D-6	D2	D4
**QF Volume (cm^3^)**	1786 (298)	1846 (283)^a^	1866 (293)^a^ ^,b^
**QF T_2_ (ms)**	32.6 (0.8)	34.5 (1.0)^a^	35.4 (2.0)^a^ ^,b^

a Significantly different from baseline (P<0.05); ^b^significantly different from D2 (P<0.05).

QF: quadriceps femoris.

### Correlations

Changes in MVC_right_ resulting from the NMES session were positively correlated with changes in 10∶100 ratio in the early phase (r = 0.43/ρ = 0.50, P<0.01). As reported in Table 4, significant correlations were found in the early phase of recovery between changes in MVC_right_ and Db_100_ (r = 0.75/ρ = 0.78, P<0.01), Db_10_ (r = 0.73/ρ = 0.79, P<0.01), RFD (r = 0.70/ρ = 0.74, P<0.01) and VAS (r = 0.37, P<0.01).

**Table 4:** Correlations matrix between changes in central and peripheral factors when pooling together the measurements performed immediately after and one day post-NMES.

	MVC_right_	VA	Db_100_	Db_10_	10∶100	TTP	RFD	VAS	CK
**MVC_right_**		ns	**0.75**/**0.78**	**0.73**/**0.79**	**0.43**/**0.50**	ns	**0.70**/**0.74**	**0.37**/ns	ns
**VA**	ns		ns	ns	ns	ns	ns	ns	ns
**Db_100_**	**0.75**/**0.78**	ns		**0.90**/**0.88**	**0.38**/**0.43**	ns	**0.96**/**0.96**	ns	ns
**Db_10_**	**0.73**/**0.79**	ns	**0.90**/**0.88**		**0.75**/**0.78**	ns	**0.85**/**0.84**	ns	ns
**10∶100**	**0.43**/**0.50**	ns	**0.38**/**0.43**	**0.75**/**0.78**		ns	0.34/**0.39**	ns	ns
**TTP**	ns	ns	ns	ns	ns		**−0.38**/ns	ns	ns
**RFD**	**0.70**/**0.74**	ns	**0.96**/**0.96**	**0.85**/**0.84**	0.34/**0.39**	**−0.38**/ns		ns	ns
**VAS**	**0.37**/ns	ns	ns	ns	ns	ns	ns		ns
**CK**	ns	ns	ns	ns	ns	ns	ns	ns	

Results are presented with Pearson r coefficient/Spearman rho rank coefficient for P<0.05. ns: non-significant (P>0.05). Results are in bold for P<0.01.

MVC_right_: right leg maximal voluntary contraction, VA: voluntary activation, Db_100_: evoked force by a doublet at 100 Hz, Db_10_: evoked force by a doublet at 10 Hz, TTP: time-to-peak force during 100 Hz doublet, RFD: maximal rate of force development during 100 Hz doublet, VAS: score in visual analog scale, CK: plasma creatine kinase activity.

In the delayed phase (i.e., D2, D3 and D4), changes in MVC_right_ were significantly correlated to changes in VA (r = 0.47/ρ = 0.49, P<0.01). As for the early phase of recovery, significant correlations were found between changes in MVC_right_ and Db_100_ (r = 0.68/ρ = 0.77, P<0.01), Db_10_ (r = 0.63/ρ = 0.74, P<0.01) and RFD (r = 0.64/ρ = 0.72, P<0.01). Additionally, changes in plasma CK activity were negatively correlated with changes in MVC_right_ (r = −0.33/ρ = −0.51, P<0.01) and, as reported in Table 5, a negative correlation was found between changes in VA and VAS (r = −0.28/ρ = −0.25, P<0.05).

**Table 5:** Correlations matrix between changes in central and peripheral factors pooling together the measurements performed two, three and four days post-NMES.

	MVC_right_	VA	Db_100_	Db_10_	10∶100	TTP	RFD	VAS	CK
**MVC_right_**		**0.47**/**0.49**	**0.68**/**0.77**	**0.63**/**0.74**	0.29/**0.32**	ns	**0.64**/**0.72**	ns	**−0.33**/**−0.51**
**VA**	**0.47**/**0.49**		**0.32**/**0.36**	**0.37**/**0.35**	0.28/ns	ns	**0.31**/**0.34**	−0.28/−0.25	ns/**−0.32**
**Db_100_**	**0.68**/**0.77**	**0.32**/**0.36**		**0.82**/**0.84**	0.26/ns	ns	**0.88**/**0.88**	ns	−0.26/**−0.38**
**Db_10_**	**0.63**/**0.74**	**0.37**/**0.35**	**0.82**/**0.84**		**0.76**/**0.68**	ns	**0.79**/**0.77**	ns/**−0.29**	−0.27/**−0.40**
**10∶100**	0.29/**0.32**	0.28/ns	0.26/ns	**0.76**/**0.68**		ns	**0.34**/ns	**−0.30**/−0.24	ns
**TTP**	ns	ns	ns	ns	ns		**−0.37**/−0.24	ns	ns
**RFD**	**0.64**/**0.72**	**0.31**/**0.34**	**0.88**/**0.88**	**0.79**/**0.77**	**0.34**/ns	**−0.37**/−0.24		ns	ns/**−0.31**
**VAS**	ns	−0.28/−0.25	ns	ns/**−0.29**	**−0.30**/−0.24	ns	ns		ns
**CK**	**−0.33**/**−0.51**	ns/**−0.32**	−0.26/**−0.38**	−0.27/**−0.40**	ns	ns	ns/**−0.31**	ns	

Results are presented with Pearson r coefficient/Spearman rho rank coefficient for P<0.05. ns: non-significant (P>0.05). Results are in bold for P<0.01.

MVC_right_: right leg maximal voluntary contraction, VA: voluntary activation, Db_100_: evoked force by a doublet at 100 Hz, Db_10_: evoked force by a doublet at 10 Hz, TTP: time-to-peak force during 100 Hz doublet, RFD: maximal rate of force development during 100 Hz doublet, VAS: score in visual analog scale, CK: plasma creatine kinase activity.

Moreover, T_2_ changes were negatively correlated with changes in MVC_right_, Db_100_ and VA (Table 6). A positive correlation was determined between changes in T_2_ and plasma CK activity at D2 (r = 0.48, P<0.05/ρ = 0.56, P<0.01) and D4 (r = 0.41, P<0.05/ρ = 0.78, P<0.01). Interestingly, changes in T_2_ at D2 were highly correlated with change in VA at D3 (r = 0.60/ρ = 0.65, P<0.01).

**Table 6:** Correlations matrix between changes in T_2_ relaxation time and central and peripheral factors.

		T_2_ relaxation time	
		D2	D4
**MVC_right_**	**After**	ns	ns
	**D1**	**−0.60**/**−0.66**	**−0.67**/**−0.75**
	**D2**	**−0.57**/**−0.72**	**−0.61**/**−0.78**
	**D3**	−0.48/**−0.59**	**−0.70**/**−0.82**
	**D4**	**−0.57**/**−0.63**	**−0.68**/**−0.82**
**VA**	**After**	ns	ns
	**D1**	**−0.52**/**−0.50**	−0.74/ns
	**D2**	**−0.63**/**−0.58**	**−0.56**/**−0.49**
	**D3**	**−0.60**/**−0.65**	**−0.71**/**−0.71**
	**D4**	ns/−0.46	ns/−0.44
**Db_100_**	**After**	ns	−0.41/−0.47
	**D1**	−0.40/−0.48	**−0.51**/**−0.60**
	**D2**	−0.40/**−0.49**	**−0.60**/**−0.71**
	**D3**	−0.40/−0.46	**−0.57**/**−0.60**
	**D4**	−0.44/**−0.51**	**−0.60**/**−0.66**
**CK**	**After**	ns	**−0.52**/ns
	**D1**	**0.55**/0.49	**0.49**/**0.57**
	**D2**	0.48/**0.56**	0.41/**0.66**
	**D3**	**0.49**/**0.59**	**0.51**/**0.77**
	**D4**	ns/**0.57**	0.41/**0.78**

Results are presented with Pearson r coefficient/Spearman rho rank coefficient for P<0.05. ns: non-significant (P>0.05). Results are in bold for P<0.01.

MVC_right_: right leg maximal voluntary isometric contraction force, VA: voluntary activation, Db_100_: evoked force by a doublet at 100 Hz, CK: plasma creatine kinase activity.

## Discussion

The major finding of the present study was that the reduction in MVC force resulting from a single bout of isometric NMES was related to both peripheral and central factors. While the early force loss was exclusively associated to peripheral factors, the prolonged force impairment was due to both peripheral and central changes.

### Early changes in neuromuscular function

In the present study, the 30% reduction in MVC force found immediately after the NMES session is similar to what has been reported in previous NMES studies using comparable stimulation intensities [10], [29]. Interestingly, the impairment of NMES-induced MVC force was related to alterations of the contractile properties as illustrated by the significant reduction of peak force for both doublets and twitch. On the contrary, the VA was not altered immediately after NMES. Our results are in accordance with two previous studies [21], [22] showing that NMES-induced muscle fatigue is due to alterations at the peripheral level. While NMES-induced neuromuscular propagation failure has been previously observed on the basis of a reduced M-wave amplitude [21], we reported, for the first time, a significant decrease of the 10∶100 ratio after isometric NMES, thereby illustrating the occurrence of a prolonged low frequency force depression [42] and suggesting an altered excitation-contraction coupling [43]. The latter result and the decrease in RFD found in our study after the NMES session could be associated to previously reported changes in intracellular energy supply, metabolite accumulation (e.g., inorganic phosphate and protons) resulting from a high metabolic demand [44] and a decrease in myofilament Ca^2+^ sensitivity [11], [12]. Thus, the immediate decrease in MVC following isometric NMES session was exclusively associated to peripheral alterations.

### Delayed effects of NMES exercise

The MVC force was still decreased by ∼20% at D4 while the CK level was 70 times higher than the baseline level thereby indicating a delayed functional effect within the days following the NMES session. Interestingly, the extent of MVC loss we measured was similar to what has been reported in previous studies [6], [9], [29]. In addition, the time course of muscle soreness and CK activity we reported was in accordance with previous studies assessing the effects of NMES-induced muscle damage with a delayed onset of muscle soreness at D2 and a peak CK activity at D4 [9], [29], [30]. However, the CK activity we measured was twice larger the level reported in a previous study using the same NMES protocol on knee extensors [10]. This larger CK increase might actually be due to the high inter-individual variability and the presence of high responders in our experimental population as previously discussed [45], [46]. Despite the large changes regarding changes in MVC and plasma CK activity, the level of muscle soreness reported by our subjects was relatively low as compared to what has been reported in previous NMES studies [9]. It has been well acknowledged that muscle soreness is a subjective variable which does not reflect the magnitude of exercise-induced muscle damage [47]. Our study provides further evidence that muscle soreness is a poor indicator of the magnitude of muscle damage as previously suggested [47], [48].

#### Peripheral contribution

MVC force loss is currently considered as the most reliable indicator of muscle injury [48]. The prolonged depression reported in the present study was associated to changes in contractile properties which led to a decrease of ∼12.5% decrease in Db_100_ and Db_10_ at D4. Interestingly, the 10∶100 ratio decreased up to one day after NMES and significantly recovered to baseline values at D2. Interestingly, two phases could be distinguished: in a first phase (i.e., at D1), the altered excitation-contraction coupling was prolonged; in a second phase, the low frequency force depression was back to normal suggesting that the altered excitation-contraction coupling mainly accounted for the decreased functional performance in an early phase. In addition, we found positive correlations between changes in MVC and the 10∶100 ratio at D1 but also with Db_100_ at D4. On that basis, peripheral alterations including muscle damage and the impaired excitation-contraction coupling (up to D1) contribute to the voluntary force loss [48] and the RFD decrease within the first four days following a single bout of isometric NMES. These results are in accordance with histological analyses showing that NMES exercise induced major muscle fibre alteration [6]–[8]. The increased CK activity, QF muscles volume and T_2_ relaxation time we reported further confirmed the prolonged peripheral alterations related to NMES. It is noteworthy that T_2_-based analysis was only performed on *quadriceps femoris* muscles. Additionally, it has been clearly established that the magnitude of muscle damage is greater when contractions are performed at long muscle length [49]. In our study, subjects were seated on a specific chair with the knee angle fixed at 100° (0° corresponding to the full extension) so that the hamstring muscles were inevitably at short muscle length. Moreover, several studies reported that muscle alterations are highly specific to the activated muscle group during the EIMD [50], [51]. For instance, increase in elbow flexors muscles T_2_ was found after EIMD whereas no change occurred in elbow extensors [50]. We quantified change in hamstring muscles T_2_ of subjects with the greatest increase in *quadriceps femoris* muscles T_2_ at D4 (*N* = 3) and a high increase in *quadriceps femoris* muscles T_2_ was found at D4 (from 32.1 (0.9) ms to 37.8 (1.4) ms) whereas change in hamstring muscles T_2_ was negligible (from 31.8 (0.7) ms to 32.0 (0.9) ms). Overall, our results clearly indicate that peripheral alterations always contribute to the NMES-induced prolonged MVC force loss.

#### Central contribution

We showed that a central activation failure illustrated by the significant decrease in VA which occurred from D2 was related to the long lasting MVC reduction. This was further supported by the positive correlation between changes in MVC and VA only in the delayed phase of recovery. On the basis of surface electromyography measurements performed at D2 after a NMES session, Laurin *et al.* (2012) reported a similar alteration in neural drive. Interestingly, the cause-effect relationship between the neural alterations and the force loss resulting from exercise-induced muscle damage seems related to the type of exercise. Indeed, several studies reported that voluntary eccentric exercise led to an immediate VA decrease and a full recovery at D2 [20], [23], [24] whereas we clearly showed that the neural alterations occurred later as a result of electrically-evoked isometric contractions. These findings might be related to the well-acknowledged non-physiological recruitment of motor units (i.e., random, spatially limited and temporally synchronous) associated to NMES [2] which, as compared to voluntary eccentric exercise, has been related to a larger amount of myofibres protein damaged and a higher inflammatory response [7]. While T_2_ has been considered as an index of inflammation within the muscle tissue [52], [53], the decrease in VA, increases in indirect marker of muscle damage (i.e., plasma CK activity, muscle soreness, muscle T_2_) and the correlation among these variables in the delayed phase of recovery might support the assumption that the activation of group III and IV afferent fibres by inflammatory mediators [19] could impair the voluntary activation of a damaged muscle [17], [18]. At the peripheral level, a potential link between alteration of muscle extracellular matrix (ECM) and neural modulation has been hypothesized [54]. On that basis, it remains to be determined whether and to what extent an alteration of ECM proteins could play a role in the neural drive impairment we evidenced after NMES EIMD.

To more deeply assess inter-relationship between peripheral and central contribution in MVC loss after NMES EIMD, it would be relevant to include additional variables accounting for central factors. Although voluntary activation is a valuable and reliable parameter used to assess non-invasively *in vivo* spinal and supraspinal contribution in voluntary force production [55], [56], magnetic stimulation of the motor cortex would provide further insights into the underlying mechanisms involved in the reduced force production resulting from NMES EIMD.

In conclusion, we showed that the immediate decrease in voluntary force production capacities after a single bout of isometric NMES was related to peripheral factors while both central and peripheral mechanisms accounted for the prolonged force reduction. We demonstrated that the contractile properties were always altered after NMES-induced muscle damage while the neural drive impairment was delayed, being significant between two and four days post-injury. Our findings indicated that the sequence of events contributing to the strength loss after a NMES session seems to differ from that occurring as a result of eccentric exercise, even though further studies would be warranted in order to carefully investigate whether the motor unit recruitment would influence the time course of neuromuscular changes induced by a damaging protocol.
